# Anatomical Posterior Acetabular Plate Versus Conventional Reconstruction Plates for Acetabular Posterior Wall Fractures: A Comparative Study

**DOI:** 10.3390/jcm13175341

**Published:** 2024-09-09

**Authors:** Chang-Han Chuang, Hao-Chun Chuang, Jou-Hua Wang, Jui-Ming Yang, Po-Ting Wu, Ming-Hsien Hu, Hong-Lin Su, Pei-Yuan Lee

**Affiliations:** 1 Department of Life Sciences, National Chung Hsing University, 402 Taichung, Taiwan; skyman889@gmail.com; 2 Doctor Program in Translational Medicine, National Chung Hsing University, 402 Taichung, Taiwan; 3 Department of Orthopaedic Surgery, Show Chwan Memorial Hospital, 500 Changhua, Taiwan; minghsienhu@gmail.com; 4 Department of Orthopaedic Surgery, National Cheng Kung University Hospital, College of Medicine, National Cheng Kung University, 704 Tainan, Taiwanajhwang15@gmail.com (J.-H.W.); anotherme500@gmail.com (P.-T.W.); 5 Department of Orthopaedic Surgery, Tainan Sin Lau Hospital, 701 Tainan, Taiwan; jmyang25@gmail.com; 6 Department of Biomedical Engineering, National Cheng Kung University, 704 Tainan, Taiwan

**Keywords:** acetabular fracture, posterior wall fracture, posterior column fracture, internal fixation, anatomical locking plate, plate osteosynthesis

## Abstract

**Background:** Functional recovery following the surgical fixation of acetabular posterior wall fractures remains a challenge. This study compares outcomes of posterior wall fracture reconstruction using an anatomical posterior acetabular plate (APAP) versus conventional reconstruction plates. **Methods:** Forty patients with acetabular fractures involving the posterior wall or column underwent surgery, with 20 treated using APAPs (APAP group) and 20 with conventional pelvic reconstruction plates (control group). Baseline patient characteristics, intraoperative blood loss and time, reduction quality, postoperative function, and postoperative complications were compared using appropriate non-parametric statistical tests. A general linear model for repeated measures analysis of variance was employed to analyze trends in functional recovery. **Results:** No significant differences were observed in baseline characteristics. APAP significantly reduced surgical time by 40 min (186.5 ± 51.0 versus 225.0 ± 47.7, *p* =0.004) and blood loss (695 ± 393 versus 930 ± 609, *p* = 0.049) compared to conventional plates. At 3 and 6 months following surgery, the APAP group exhibited higher functional scores (modified Merle d’Aubigné scores 10 ± 1.8 versus 7.8 ± 1.4, *p* < 0.001; 13.4 ± 2.8 versus 10.1 ± 2.1, *p* = 0.001), converging with the control group by 12 months (modified Merle d’Aubigné scores 14.2 ± 2.6 versus 12.7 ± 2.6, *p* = 0.072; OHS 31.6 ± 12.3 versus 30.3 ± 10.1, *p* = 0.398). Radiologically, the APAP group demonstrated superior outcomes (*p* = 0.047). Complication and conversion rates to hip arthroplasty did not significantly differ between groups (10% versus 15%, *p* = 0.633). **Conclusions**: The use of an APAP in reconstructing the posterior acetabulum significantly reduces surgical time, decreases intraoperative blood loss, and leads to earlier functional recovery compared to conventional reconstruction plates. The APAP provides stable fixation of the posterior wall and ensures the durable maintenance of reduction, ultimately yielding favorable surgical outcomes.

## 1. Introduction

Fractures of the posterior wall are the most common type of acetabular fractures, accounting for nearly a third of all fractures of the acetabulum [1,2]. Plate osteosynthesis is widely acknowledged as the preferred method for treating specific types of acetabular fractures involving the posterior column or posterior wall [3,4]. Achieving anatomical reduction and ensuring stable fixation are imperative to prevent posttraumatic osteoarthritis (PTOA), osteonecrosis of the femoral head (ONFH), and potential progression to total hip arthroplasty (THA) [5,6,7]. While restoring acetabular congruence is essential for functional recovery [8], managing fractures of the posterior column or wall presents significant challenges, particularly for less experienced surgeons, due to the complex anatomy of the acetabulum and the frequent occurrence of concomitant hip dislocation [9,10]. A user-friendly internal fixation device would be immensely beneficial in these cases.

Numerous fixation techniques have been proposed, including single plating, dual plating, and fragment-specific fixation [11,12,13]. However, the complex bone structure often necessitates the manual contouring of plates to conform to the curvature. The intraoperative bending and shaping of plates can be time-intensive and imprecise, potentially compromising their mechanical integrity [14]. Additionally, addressing comminuted wall fractures poses another obstacle. While Ritcher et al. introduced the concept of spring plates beneath a buttress plate, the technique is intricate and carries a risk of articular surface damage and challenges in plate positioning [15]. The previous literature has described a few anatomical plates, including W-shaped and H-shaped acetabular angular plates, designed to reconstruct posterior wall fractures and reduce intra-articular screw penetration [16,17]. While these plates demonstrated a lower penetration rate on immediate postoperative radiographs, their impact on functional recovery compared to that in a control group was not thoroughly explored [16].

This study aims to investigate the efficacy of using an anatomical posterior acetabular plate (APAP, produced by INTAI Technology Corp., Taichung, Taiwan) in promoting functional recovery, reducing surgical time, and preventing complications. To quantitatively assess the clinical utility of the APAP, we compared the outcomes of a patient cohort treated with APAPs to those of a separate group treated with conventional reconstruction plates.

## 2. Materials and Methods

### 2.1. Population 

**Figure 1:**
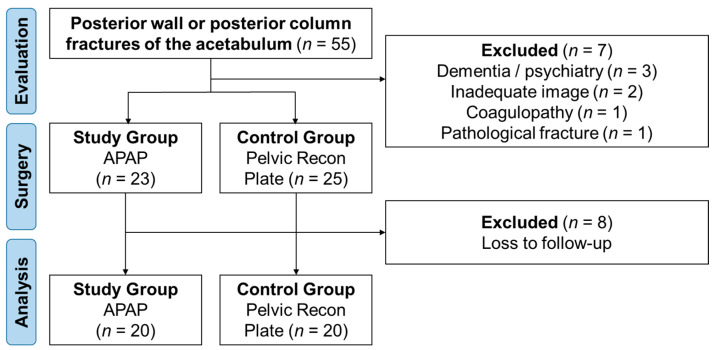
Patient inclusion flow chart.

From January 2015 to December 2018, acetabular fractures treated by the senior author at a single level-I trauma center were retrospectively reviewed. Patients with posterior wall or posterior column fractures of the acetabulum requiring open reduction and internal fixation (ORIF) were included. Surgical indications encompassed hip instability, hip joint incongruity with an articular step-off exceeding 2 mm, inadequate secondary congruence, the presence of intra-articular fragments, and posterior column displacement exceeding 2 mm. Patients underwent treatment with either the APAP or a pelvic reconstruction plate. All implants used were FDA approved. Exclusion criteria comprised pathological acetabular fractures, neuropathic arthropathy, coagulopathy, dementia, and other conditions affecting postoperative compliance. (see Figure 1)

### 2.2. Radiographic Evaluation 

A standardized preoperative radiographic imaging protocol included standard anteroposterior pelvic films, two 45° oblique Judet views, and pelvic inlet and outlet views. Three-dimensional (3D) computed tomography (CT) images (slice thickness: 3 mm) and reconstructed pelvic images were obtained to enhance surgical planning. For patients with dislocation, attempted closed reduction and skin traction preceded the radiographic protocol.

### 2.3. Implant Design

**Figure 2:**
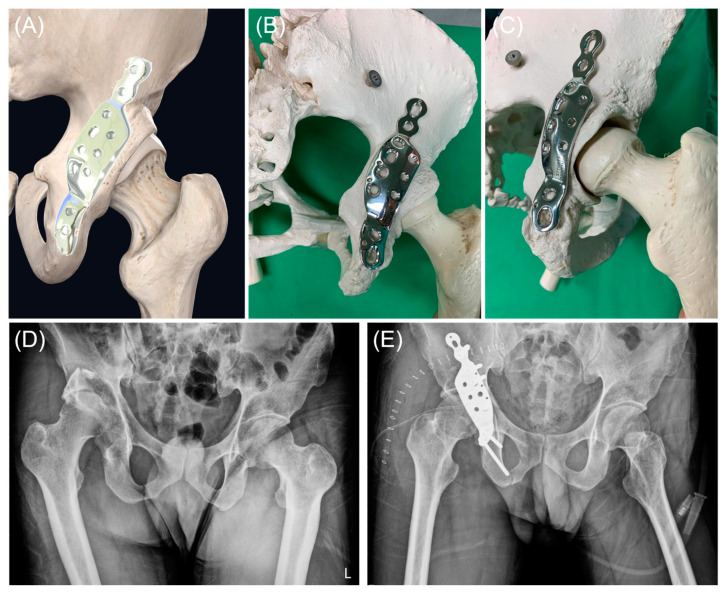
Design and application of the anatomical posterior acetabular plate (APAP). (**A**) A 3D reconstruction of the right hip joint demonstrates the APAP’s design and optimal positioning for plating the posterior wall of the acetabulum. (**B**,**C**) Application of the APAP on a sawbone model in posteroanterior and iliac oblique views, respectively. (**D**) Radiograph displaying a case with a posterior wall fracture and concurrent posterior hip dislocation. (**E**) Postoperative radiograph illustrates the case after open reduction and internal fixation with the APAP, achieving anatomical reduction and concentric alignment of the hip joint.

The APAP (see Figure 2) was tailored to fit the structure of the posterior column of the acetabulum in the Taiwanese population. Utilizing a series of non-contrast pelvic CT images from our hospital’s database, a 3D pelvic reconstruction model was created through segmentation using the marching cubes algorithm. The APAP was made from 18Chromium-14Nickel-2.5Molybdenum stainless steel, also known as AISI 316LVM. This stainless steel is vacuum melted to achieve the high levels of purity and cleanliness required for surgical implants. The plate is manufactured in accordance with the ASTM F139-19 Standard Specification for Wrought 18Chromium-14Nickel-2.5Molybdenum Stainless Steel Sheet and Strip for Surgical Implants (UNS S31673). The APAP was produced by INTAI Technology Corp., Taichung, Taiwan.

The APAP comprises three components: the iliac, acetabular, and ischial components. The iliac and ischial components feature one locking hole and one compression hole (Figure 2A). The acetabular component incorporates anterior and posterior rows for addressing the posterior column and posterior wall, respectively. The anterior row accommodates either a 4.5 mm compression screw or a 4.5 mm locking screw. The posterior row has a fixed angle and utilizes 3.5 mm locking screws to prevent intra-articular screw penetration. The contour of the APAP is derived from 3D pelvic reconstructions. The acetabular component is slightly underbent to induce a tension band effect. Two sizes, standard and narrow, are available to accommodate variations in the distance between the sciatic notch and the rim of the posterior wall.

### 2.4. Surgical Procedure

All patients underwent general anesthesia and were positioned prone on a radiolucent table. The standard Kocher–Langenbeck approach was employed to visualize the fracture site, with the hip extended and the knee flexed beyond 90 degrees to facilitate sciatic nerve retraction. A Schanz screw was positioned over the trochanteric region to aid in manual distraction for hip distraction and improved joint visualization. The articular surface was exposed, and incarcerated fragments were elevated until flush with the articular surface. In fractures involving both the posterior column and posterior wall, posterior column reduction preceded posterior wall reduction. Farabeuf clamps or reduction clamps were utilized for fracture reduction, and a Schanz screw was inserted into the ischial tuberosity to serve as a joystick. Preliminary fixation was achieved using Kirschner wires (K-wires) or lag screws following posterior column reduction. Marginal impaction was corrected and temporarily fixed with K-wires.

Patients in the study group were treated with the APAP, applied along the curvature of the ilium, acetabulum, and ischium. Patients in the control group received treatment with a pelvic reconstruction plate. After provisionally fixing the appropriate plate holes, the quality of fracture reduction and implant positioning were assessed under fluoroscopic guidance. Intraoperative fluoroscopic checks included anteroposterior and obturator oblique views of the hip, axial views of the screws, and tangential views of the screws.

### 2.5. Postoperative Follow-Up and Rehabilitation Protocol

All patients received routine intravenous antibiotics, initiated during anesthesia induction and continued for one day post-surgery. Passive hip mobilization was encouraged on the first postoperative day, with active hip movement encouraged on the second postoperative day. Hip precautions, restricting flexion to less than 90° and preventing adduction, were implemented for the first 6 weeks. Non-weight-bearing ambulation was advised for 4–6 weeks to prevent the early loss of reduction. Patients progressed to full weight bearing only after radiographic and clinical confirmation of fracture union.

### 2.6. Outcome Measurements

Preoperative patient characteristics, encompassing age, gender, fracture pattern, presence of sciatic nerve injury, marginal impaction, and hip fracture/dislocation, were documented. Surgical details, such as intraoperative blood loss and duration of surgery, were also recorded. After surgery, patients underwent regular follow-ups at the outpatient department at intervals of 1, 2, 3, 6, and 12 months, followed by annual visits. All patients were followed up for a minimum of 2 years. During each follow-up, clinical functional recovery was semi-quantitatively assessed using the modified Merle d’Aubigné scoring system. At the final follow-up, clinical evaluation was conducted using the Oxford Hip Score (OHS) questionnaire [18], while radiological evaluation was performed according to Matta’s criteria [1,19]. Utilizing posteroanterior and oblique pelvic X-rays, displacement was categorized as anatomical (0–1 mm), successful (2–3 mm), or poor (>3 mm). Recorded complications included postoperative sciatic nerve palsy, infection, heterotopic ossification (HO), osteonecrosis of the femoral head (ONFH), hip redislocation, implant failure, and the necessity for conversion to total hip arthroplasty (THA).

### 2.7. Statistical Analysis

To compare baseline characteristics between the study group and the control group, the non-parametric Mann–Whitney U test was utilized for continuous variables, while the Chi-squared test was employed for categorical variables. Functional recovery over time was examined using a general linear model for repeated measures analysis of variance. The cohort was divided based on the implants utilized, and measurements were taken at postoperative intervals of 1, 2, 3, 6, and 12 months. Differences in trends over the year between subgroups was assessed using between-subject effects. The Mann–Whitney U test was employed for the comparison of functional scores at specific time points. All statistical analyses were conducted using SPSS software (version 17.0; IBM, Armonk, NY, USA), with the significance level set at *p*  <  0.05.

## 3. Results

### 3.1. Patient Characteristics 

A total of 40 patients meeting the inclusion criteria were enrolled, with an average follow-up duration of 27.8 months (range, 24–60 months). The study group comprised 20 patients treated with APAP, while the control group consisted of 20 patients treated with a pelvic reconstruction plate. There were no statistically significant differences between the two groups in terms of gender distribution, acetabular fracture pattern based on Judet and Letournel classification, preoperative dislocation rate, time to definitive ORIF, and duration of final follow-up (*p* = 0.212) (Table 1). Motor vehicle accidents were the leading cause of injury, accounting for 90% (36 patients), while falls from height constituted the remaining 10% (4 patients) of cases. 

A comparison of operative times between the two groups revealed a statistically significant difference (186.5 min versus 225 min, *p* = 0.004), indicating that APAP saved approximately 40 min of surgical time. Additionally, blood loss was significantly lower in the study group compared to that in the control group (695 mL versus 930 mL, *p* = 0.049).

### 3.2. Functional and Radiological Outcomes 

**Figure 3:**
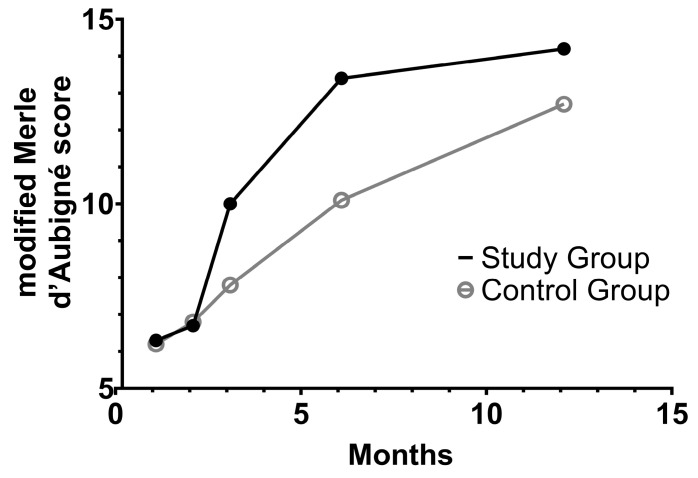
Functional recovery following open reduction and internal fixation of acetabular fractures involving the posterior wall. Patients treated with the anatomical posterior acetabular plate were placed into the study group (black dots), while those with conventional pelvic reconstruction formed the control group (hollow circles). A statistically significant difference in functional recovery emerged (*p* = 0.007, determined through tests of between-subjects effects of repeated measures ANOVA). At 3 and 6 months following surgery, the modified Merle d’Aubigné scores were significantly higher in the study group (*p* < 0.001 and *p* = 0.001, respectively; determined through Mann–Whitney U tests). However, by the 12-month mark, there was no statistically significant difference between the two groups (*p* = 0.072).

Upon stratifying the patients into two groups based on the implants used, notable trends in functional recovery emerged, with a statistically significant difference observed (*p* = 0.007, determined through tests of between-subjects effects of repeated measure ANOVA) (see Figure 3). At 3 and 6 months following surgery, the modified Merle d’Aubigné scores in the two groups showed statistically significant differences (10 ± 1.8 versus 7.8 ± 1.4, *p* < 0.001; 13.4 ± 2.8 versus 10.1 ± 2.1, *p* = 0.001, determined through Mann–Whitney U tests). However, by the 12-month mark, functional recovery appeared to converge once again, with no statistically significant differences observed in the modified Merle d’Aubigné scores (14.2 ± 2.6 versus 12.7 ± 2.6, *p* = 0.072) or the OHS (31.6 ± 12.3 versus 30.3 ± 10.1, *p* = 0.398). 

Likewise, the radiological outcomes assessed using Matta’s criteria at 12 months after surgery did not demonstrate significant differences between the study and control groups (*p* = 0.204, determined through Chi-squared analysis). The radiological outcomes were significantly superior in the study group (*p* = 0.047). Anatomical reduction was sustained in 70% of the study group compared to 35% of the control group, while successful reduction was maintained in 15% of the study group and 50% of the control group (Table 2).

### 3.3. Complications 

**Figure 4:**
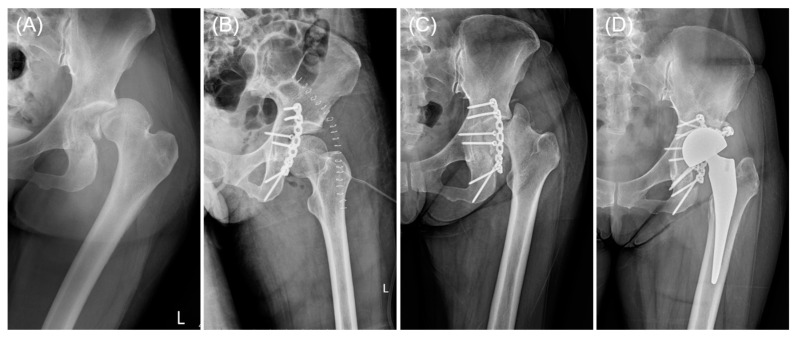
A case of acetabular fracture treated with conventional pelvic reconstruction plate complicated by recurrent dislocation. (**A**) The patient sustained concurrent left acetabular posterior wall fracture and posterior dislocation of the left hip joint. (**B**) The patient underwent open reduction and internal fixation with a conventional pelvic reconstruction plate. (**C**) Eight months after the index surgery, the patient experienced recurrent hip dislocation, accompanied by a concurrent femoral head fracture. (**D**) Eventually, the patient underwent secondary surgery and conversion to bipolar hemiarthroplasty.

Postoperative complications are outlined in Table 3. Among patients treated with APAP (the study group), foot drop was noted in two cases: one had preoperative foot drop, which resolved by 3 months after surgery, while the other experienced transient postoperative foot drop, spontaneously resolving within 2 months after surgery. Similarly, two patients in the control group developed postoperative foot drop, with both cases resolving by 6 months after surgery. One patient treated with a conventional pelvic reconstruction plate (the control group) experienced recurrent hip dislocation, resulting in the fracture and fragmentation of the osteonecrotic femoral head, ultimately necessitating conversion to hip arthroplasty (see Figure 4). Notably, none of the three patients in the study group who suffered from postoperative recurrent dislocation experienced femoral head fracture. The rate of conversion to hip arthroplasty did not differ significantly between the study and control groups (10% versus 15%, *p* = 0.633). Postoperative infection rates did not differ significantly between the two groups (5% vs. 10%, *p* = 0.548). Among the patients treated with the conventional pelvic reconstruction plate, one developed a deep infection requiring debridement and implant retention, while the other two experienced superficial infections and cellulitis, both of which were successfully managed with intravenous antibiotics. Additionally, the prevalence of other complications, including osteoarthritis, osteonecrosis of the femoral head, and heterotopic ossification, did not vary between the two groups. (Table 3)

## 4. Discussion

The management of posterior wall or posterior column fractures presents significant challenges due to the critical need for achieving both anatomical reduction and stable fixation, which is inherently difficult to accomplish [20]. This study introduces a novel APAP, which not only eliminates the need for custom bending and reduces intraoperative blood loss but also enhances stability, leading to expedited functional recovery compared to conventional pelvic reconstruction plates. The design of the APAP provides orthopedic trauma surgeons with a durable and time-efficient alternative, paving the way for future advancements in anatomical acetabular plates.

Traditionally, posterior plating for acetabular fractures involved the utilization of rim plates, buttress plates, or spring plates, either individually or in various combinations [12,13,15]. However, these methods are time-consuming and technically demanding. Additionally, repeated contouring of the reconstruction plate could potentially compromise its inherent mechanical strength [14,21]. The past literature has introduced a few anatomical acetabular locking plates, highlighting the biomechanical advantages of such plates over conventional reconstruction plates [22]. For instance, Zhang et al. developed a W-shaped acetabular angular plate (WAAP) for reconstructing acetabular posterior wall fractures [16]. In a retrospective comparison with conventional reconstruction plates, the WAAP effectively reduced intra-articular screw penetration under intraoperative fluoroscopy. However, functional recovery trends between the WAAP group and the conventional reconstruction plate group were not compared. Similarly, Huang et al. proposed an H-shaped anatomical titanium plate for posterior plating in acetabular fractures [17]. Although the authors reported satisfactory radiological and functional outcomes with low complication rates, the study was descriptive and lacked a control group. Other studies have explored the use of 3D printed patient-specific plates [23,24,25]. Despite promising outcomes, 3D printing technology remains costly and may not be readily available in general hospitals. In contrast, the APAP is a commercialized anatomical locking plate, proven to be a cost-effective and safe alternative to conventional reconstruction plates in the current study. Moreover, the APAP offers an additional ischial component for fixation compared to the plates designed by Zhang et al. and Huang et al. [16,17]. The design of the APAP was intended to replicate the biomechanical advantages of a double plate by incorporating two rows of holes for locking screws. This, combined with the broader width and increased screw count, enhances the rigidity and stability of the construct. These features may explain the slight advantage observed in terms of improved reduction after 12 months. The APAP costs approximately $2500, compared to around $2000 for the low-profile pelvic system. In cases where dual pelvic reconstruction plates are required for treating posterior wall or posterior column fractures, the APAP proves to be economically advantageous, as it requires only a single plate. In summary, the APAP is demonstrated to be a safe, effective, and more versatile option in managing comminuted posterior wall fractures. 

Safety and efficacy are the primary goals in the internal fixation of posterior acetabular fractures. Early (<48 h) and delayed surgeries have been extensively studied, with earlier intervention recommended for relatively simple fracture patterns [26], while the timing for complex fractures remains controversial [27]. Complex fracture patterns also tend to result in greater blood loss during surgery compared to posterior wall fractures [27]. Some studies have suggested that combining epidural and general anesthesia can help reduce blood loss [28]. A positive correlation between surgical time and blood loss has been consistently observed in the literature [27,29,30]. In the current study, the use of the anatomical posterior acetabular plate (APAP) shortened surgical time by eliminating the need for bending the plate and simplifying the templating process. Additionally, the anatomical design of the APAP reduced the likelihood of intra-articular screw penetration, thereby minimizing the need for screw adjustments [16]. Less adjustment and less extensive soft tissue dissection, which is possible with a plate that conforms more closely to the native anatomy, thereby reduced the surgical time and associated bleeding. These advantages are particularly beneficial for managing complex posterior acetabular fractures and for surgeons who are in the early stages of treating these fractures.

Acetabular fractures often result in long-term morbidity, with variable trajectories of functional recovery. Letournel reported that despite achieving 94% perfect reductions of posterior wall fractures, only 79.5% of cases attained at least a very good result [10]. This discrepancy was attributed by Letournel et al. to associated osteonecrosis and comminution of the posterior wall [10,31]. Similarly, Matta et al. found slightly inferior outcomes in acetabular fractures involving the posterior wall in their investigation of 20-year survivorship in 816 patients following open reduction and internal fixation of displaced acetabular fractures [32]. The 20-year survivorship of simple posterior wall fractures was 76%, while that of associated posterior column and posterior wall fractures was 85% [32]. Recently, Tucker et al. discussed the recovery trajectory of surgically treated acetabular fractures [33]. Between six months and one year postoperatively, only 37.3% of patients reached the minimal clinically important difference (MCID), and a significant proportion (38.1%) failed to achieve the MCID even after five years [33]. The ratio of anatomical reduction was 70%, or 85% if acceptable alignment was also included, consistent with previous literature. In the current study, the APAP facilitated short-term functional recovery by approximately three months compared with conventional pelvic reconstruction plates. Remarkably, most patients in our study presented with posterior wall involvement, which typically carries a poorer prognosis. The improved functional outcomes in the APAP group may be attributed to several factors. The anatomical design of the plate likely resulted in less soft tissue dissection, reducing surgical trauma and promoting quicker recovery. Additionally, the precise anatomical fit of the APAP provided more stable fixation and better initial alignment, creating a more favorable environment for healing and rehabilitation. The locking screws in the specially designed ischial limb also contributed to enhanced stability. In summary, the APAP shows promise in the treatment of complex acetabular fractures involving the posterior wall and has the potential to hasten postoperative functional recovery. 

Late complications of posterior acetabular fractures, such as end-stage hip osteoarthritis, osteonecrosis of the femoral head, or heterotopic ossification, often necessitate conversion to total hip arthroplasty [20]. The conversion rate to hip arthroplasty varies widely. The anatomical restoration of the acetabulum is considered the most critical step in preventing these late complications and eventual conversion to hip arthroplasty [34]. Maintaining anatomical reduction has been linked to favorable functional and radiological outcomes, with reported rates of 71–86.4% and 17–95% in patients, respectively [3,35,36,37,38]. Dunet et al. reported a conversion rate of 34.7% over a 10-year period [5], while Cichos et al. reported a conversion rate of 16%, with 52% of conversions occurring within 1 year post-surgery [39]. Similarly, Firoozabadi et al. evaluated 65 patients with posterior wall acetabular fractures treated with ORIF, reporting a 17% conversion rate after 9 years [40]. In our study, the conversion rate to total hip arthroplasty was 15% in patients treated with conventional pelvic reconstruction plates after 4 years of follow-up, consistent with the previous literature. Among patients treated with APAP, the conversion rate was approximately 10%. The relatively low mid-term conversion rate in the APAP group may be attributed to the more stable fixation of the plate and better maintenance of anatomical reduction due to its anatomical design after 4 years. 

Several limitations of this study should be acknowledged. First, the sample size was relatively small, as this was a single-institution study, which may limit the statistical power and generalizability of the findings. Additionally, patients who did not complete at least 2 years of follow-up were excluded. Second, the study design was retrospective, which may have introduced bias despite efforts to control for baseline characteristics between the study group and the control group. Third, biomechanical features and fatigue analyses were not included in the current study; thus, a quantitative assessment of the rigidity and efficacy of buttressing was not possible. Fourth, the medium-term follow-up period (mean of 27.8 months) may not fully capture long-term differences in functional recovery and complications. We plan to conduct further analysis after the patients complete a 5-year follow-up. Finally, the potential influence of surgical experience is another limitation, as the learning effect could not be fully accounted for in this retrospective design. More experienced surgeons may achieve shorter surgical times, which could influence the study outcomes. The APAP was not applicable for some more complex acetabular fractures. In other words, the benefits of the APAP may be more pronounced for less experienced surgeons compared to their more experienced counterparts. These limitations warrant cautious interpretation of the study findings and highlight areas for further research.

## 5. Conclusions

The use of an APAP in reconstructing the posterior acetabulum significantly reduces surgical time, decreases intraoperative blood loss, and leads to earlier functional recovery compared to conventional reconstruction plates. The APAP provides stable fixation of the posterior wall and ensures the durable maintenance of reduction, ultimately yielding favorable surgical outcomes.

## Figures and Tables

**Table 1:** Patient characteristics.

		Study Group (*n* = 20)		Control Group (*n* = 20)		
Category	Subcategory	Count	Percentage	Count	Percentage	*p* Value
Gender	Male	18	90%	15	75%	0.212
	Female	2	10%	5	25%	
Fracture pattern	PW	7	35%	13	65%	0.088
	PCPW	4	20%	3	15%	
	TPW	4	20%	0	0%	
	BC	3	15%	4	20%	
	ACPH	2	10%	0	0%	
Preoperative dislocation	Present	8	40%	6	30%	0.504
	Absent	12	60%	14	70%	
Age		45.7	16.7	45.3	19.8	0.947
BMI		22.7	2.4	23.3	3.4	0.574
Time to ORIF (days)		4.2	3.2	4.1	2.5	0.820
Operative time (minutes)		186.5	51.0	225.0	47.7	0.004
Blood loss (mL)		695	393	930	609	0.049
Follow-up period (months)		40.6	11.7	34.9	8.8	0.121

The study group comprised 20 patients treated with an anatomical posterior acetabular plate (APAP), while the control group consisted of 20 patients treated with pelvic reconstruction plates. The *p* value was calculated using the Mann–Whitney U test for continuous variables and Chi-squared tests for categorical variables. (SD, standard deviation; PW, posterior wall; PCPW, posterior column and posterior wall; TPW, transverse and posterior wall; BC, both column; ACPH, anterior column and posterior hemi-transverse; BMI, body mass index; ORIF, open reduction and internal fixation).

**Table 2:** Radiological outcomes of acetabular treatment.

Matta Criteria	Study Group	Control Group	*p* Value
Anatomical	14	7	0.047
Successful	3	10	
Poor	3	3	

The study group comprised 20 patients treated with an anatomical posterior acetabular plate (APAP), while the control group consisted of 20 patients treated with pelvic reconstruction plates. *p* value was generated using a Chi-squared test.

**Table 3:** Complications rates after open reduction and internal fixation of acetabular fracture.

	Study Group	Control Group	*p* Value
Foot drop	10% (2/20)	10% (2/20)	1.00
Recurrent dislocation	15% (3/20)	10% (2/20)	0.633
Infection	5% (1/20)	10% (2/20)	0.548
Osteoarthritis	35% (7/20)	30% (6/20)	0.736
Osteonecrosis of femoral head	25% (5/20)	20% (4/20)	0.705
Heterotopic ossification	5% (1/20)	10% (2/20)	0.548

The study group comprised 20 patients treated with an anatomical posterior acetabular plate (APAP), while the control group consisted of 20 patients treated with pelvic reconstruction plates. The *p* value was generated using Chi-squared analysis.

## Data Availability

The data are available on reasonable request.
